# Functional Polymorphism in the *ADRB3* Gene, Encoding the Beta-3 Adrenergic Receptor, and Response to Intra-Detrusor Injection of Botulinum Toxin-A in Women with Overactive Bladder

**DOI:** 10.3390/jcm11247491

**Published:** 2022-12-17

**Authors:** Sylwester Michał Ciećwież, Klaudyna Lewandowska, Agnieszka Boroń, Jacek Brodowski, Jacek Kociszewski, Jeremy Simon Clark, Andrzej Ciechanowicz

**Affiliations:** 1 Department of Gynecology, Endocrinology and Gynecological Oncology, Pomeranian Medical University, 71-252 Szczecin, Poland; 2 Department of Clinical and Molecular Biochemistry, Pomeranian Medical University, 70-111 Szczecin, Poland; 3 Department of Primary Health Care, Pomeranian Medical University, 70-204 Szczecin, Poland; 4 Department of Gynecology, Evangelisches Krankenhaus Hagen, 58135 Hagen, Germany

**Keywords:** beta-3 adrenoreceptor, botulinum neurotoxin-A, gene polymorphism, overactive bladder

## Abstract

Background: There are reports suggesting an association between the rs4994 polymorphism in the *ADRB3* gene encoding the beta-3 adrenergic receptor and OAB risk in females. The injection of botulinum toxin-A into the bladder wall is recommended as a possible treatment for OAB patients in whom first-line therapies have failed. The aim of our study was to analyze the possible association between the *ADRB3*:rs4994 polymorphism and the patient-perceived response to a single intra-detrusor injection of botulinum toxin-A in Polish women with overactive bladder. Methods: The study group consisted of 115 consecutive female patients with OAB. The response to botulinum toxin-A was evaluated at three months after injection, as absolute or relative reductions in OAB symptoms or in scores from questionnaires ICIQ-OAB (parts A and B) and ICIQ-LUTS-QoL (parts A and B). *ADRB3*:rs4994 variants were identified by the sequencing of genomic DNA extracted from buccal swabs. Results: There were no statistically significant differences between *ADRB3*:rs4994 [T];[T] homozygotes and [T];[C]+[C];[C] subjects for absolute or relative reductions in symptoms or in scores from all four questionnaire parts at three months after the injection of botulinum toxin-A. Conclusions: Our results do not support the hypothesis that *ADRB3*:rs4994 polymorphism is associated with the response to the intra-detrusor injection of botulinum toxin-A in Polish females with overactive bladder.

## 1. Introduction

Overactive bladder syndrome (OAB) is defined by the International Continence Society (ICS) as urinary urgency, usually with urinary frequency and nocturia, with or without urgency urinary incontinence [1,2]. Ethans et al. indicated that OAB can be further differentiated into cases having neurological causes (mostly multiple sclerosis) or as being idiopathic [3].

Results recently reviewed by Piętak and Rechberger provided strong evidence that an imbalance between sympathetic and parasympathetic activity can lead to homeostatic disruption and the subsequent development of OAB [4], and studies by Hannestad et al. [5] and Rohr et al. [6] indicated that urinary incontinence has a hereditary component.

Sympathetic nerve activity contributes to urine storage by relaxing the detrusor muscle via beta-adrenoreceptors (=beta adrenergic receptors; -ARs), the most important -AR involved in bladder relaxation during the storage phase of the micturition cycle being the beta-3 adrenergic receptor [7,8,9].

The human beta-3 adrenergic receptor is encoded by the *ADRB3* gene located at chromosome 8p11.23. The *ADRB3* polymorphism with the highest frequency of minor alleles is the rs4994:c.190T>C transition, which causes the substitution of tryptophan (W) by arginine (R) at amino acid position 64 (p.Trp64Arg = p.W64R). The p.64Arg variant seems to result in the biochemical dysfunction of the receptor by reducing its activation [9,10,11]. However, as recently reviewed by Michel, the available evidence of p.64Arg hypofunctionality is largely limited to in vitro studies [12].

In 2011, Ferreira et al. provided evidence that the *ADRB3:*rs4994 polymorphism might be associated with the prevalence of OAB syndrome in Brazilian women [9]. Three years later, Honda et al. provided evidence for an association of the *ADRB3*:c.190C allele with a predisposition for overactive bladder in Japanese females [10]. However, other authors have reported conflicting results [13,14]. Concerning severity, the results of a study carried out on 1015 Dutch male patients, which focused more on lower urinary tract symptoms than on the presence of OAB syndrome, did not support a hypothesis that the *ADRB3*:rs4994 polymorphism is associated with the severity of changes in bladder function (rather than with OAB risk) [15]. Little information is available on the responses to a therapeutic agent in relation to this polymorphism.

Many patients suffering from OAB do not respond to therapies such as anticholinergic treatment, lifestyle modification, bladder retraining, or pelvic floor exercises. Botulinum toxin type A (toxin-A), a very potent neurotoxin originally from the bacterial genus *Clostridium*, has recently been recommended in guidelines as a second- or third-line treatment. The injection of botulinum toxin-A into the bladder wall may offer a treatment option for patients with proven detrusor overactivity in whom first-line therapies have failed [16]. Treatment with botulinum toxin could be a reasonable alternative to neural stimulation and has only limited side effects, generally related to the surgical procedure [17]. Recently, Ciofu et al. revealed that the response to botulinum toxin in patients with neurogenic detrusor overactivity can be improved by additional treatment with solifenacin, a selective antagonist of the M3 muscarinic receptor [18]. Therefore, we decided to analyze the possible association between the *ADRB3*:rs4994 polymorphism and response at three months to a single intra-detrusor injection of botulinum toxin-A in Polish women with overactive bladder.

## 2. Materials and Methods

### 2.1. Female Patients with Overactive Bladder

All patients gave informed, written consent to participate in the study, which was approved by the bioethics committee at the Pomeranian Medical University (PUM) in Szczecin, West Pomerania, Poland (decision no. KB-0012/125/17; 16 November 2017). The study group consisted of 115 consecutive women with OAB (from 22 to 86 years old), diagnosed using the International Consultation on Incontinence Questionnaire for OAB (ICIQ-OAB) at the Department of Gynecology, Endocrinology, and Gynecological Oncology, PUM. All patients in the study were Polish women of European descent living in West Pomerania. Inclusion criteria were: detrusor overactivity confirmed by urodynamics testing, and intolerance to or ineffective pharmacological treatment of OAB. Exclusion criteria included: previous use of botulinum toxin-A; mixed urinary incontinence; presence of urinary tract infection; bladder stones; bladder cancer; urinary retention; and/or previous urogynecological surgeries. Basic characteristics of the female OAB patients assessed included: age; body height; body mass; body mass index (BMI), calculated as body mass (kg)/height (m^2^); number of pregnancies; number of deliveries; and number of cesarean sections.

Prior to the injection of botulinum toxin-A, a buccal swab was taken from every OAB female for the extraction of genomic DNA. One hundred units of botulinum toxin-A (Botox^®^, Allergan Inc., Irvine, CA, USA) were diluted in 10 mL of normal saline (0.9% NaCl). All injection procedures were performed by a single urogynecologist (S.C.) with general anesthesia. A rigid cystoscope and needle (injeTak^®^, Laborie, Portsmouth, NH, USA) were used to inject botulinum toxin-A into the detrusor muscle at 20 sites (5 U of botulinum toxin-A per site) spaced approximately evenly over the bladder, including the dome but sparing the trigone.

All patients reported frequencies for urgency, urgency incontinence, frequency and nocturia, and completed a survey with the ICIQ-OAB questionnaire and the International Consultation on Incontinence Questionnaire-Lower Urinary Tract Symptoms-Quality of Life (ICIQ-LUTS-QoL) questionnaire, just before and three months after the intra-detrusor injection of botulinum toxin-A. In both questionnaires, part A assessed symptom severity, and part B assessed accumulative anxiety in the patient. High scores in the questionnaires suggest worse symptom profiles (ICIQ-OAB) and/or a negative impact on the quality of life (ICIQ-LUTS-QoL) [19,20]. With ICIQ-OAB, the total raw scores in part A ranged from 0 to 16 points, and in part B, from 0 to 40 points. With ICIQ-LUTS-QoL, the total raw scores in part A ranged from 0 to 76 points, and in part B, from 0 to 190 points. In addition, absolute (Δ_0–3_ = frequency or score before injection minus that at 3 months after injection) or relative (Δ_%_ = frequency or score before injection minus that at 3 months after injection)/frequency or score before injection) reductions in symptom frequencies or scores were calculated for each part of each questionnaire. 

### 2.2. Genotyping

Genomic DNA was extracted from buccal epithelial cells using a commercially available DNA isolation kit (PrepFiler^®^ Express Forensic DNA Extraction Kit, Applied Biosystems, Life Technologies Polska, Warsaw, Poland) according to the manufacturer’s instructions. The amplification of the *ADRB3* sequence of 440 base pairs (bp) in length, including the rs4994 polymorphism, was performed by PCR, using 5′-CGCCCAATACCGCCAACA CCAGT-3′ as the forward primer and 5′-CGCGGCCGACACGACCCACAC-3′ as the reverse primer. Subsequently, the PCR amplification products were purified using Exonuclease I and FastAP Thermosensitive Alkaline Phosphatase (ThermoFisher Scientific Inc., Waltham, MA, USA) according to manufacturer procedures. The sequencing of the products used BigDye^®^ Terminator v3.1 Cycle Sequencing Kits (Applied Biosystems, Life Technologies Polska, Warsaw, Poland). Electrophoresis and analyses were performed according to manufacturer procedures, using an ABI PRISM 3100-Avant machine (Data Collection Software v2.0 and Sequencing Analysis Software v5.4; Applied Biosystems).

### 2.3. Statistical Analyses

Deviations from normality for the distributions of quantitative data were tested using Shapiro–Wilk tests. Since the majority of quantitative variables were not normally distributed, all are presented as medians with minimum and maximum values. Quantitative data were compared between genotype groups using Mann–Whitney tests. Categorical data and the divergence of *ADRB3*:rs4994 genotype frequencies from Hardy–Weinberg equilibria were assessed using chi-squared or Fisher’s exact tests. Statistical significance was defined as *p* < 0.05. All data were analyzed using a data analysis software system (Dell Statistica, version 13. Dell Inc. 2016, software.dell.com, accessed on 3 April 2022).

## 3. Results

There were 105 [T];[T] homozygotes (91.3%), 7 [T];[C] heterozygotes (6.1%), and 3 [C];[C] homozygotes (2.6%) (referred to as TT, TC, and CC in this article) in the studied group of women with overactive bladder. The frequency of the minor ADRB3:c.190C allele was 5.6%. In the subgroup of women with idiopathic OAB (iOAB), there were 72 TT homozygotes (90.0%), 5 TC heterozygotes (6.2%), and 3 CC homozygotes (3.8%), and the frequency of the minor ADRB3:c.190C allele was 6.9%. In the subgroup of women with neurogenic OAB (nOAB), there were 33 TT homozygotes (94.3%) and 2 TC heterozygotes (5.7%), and the frequency of the minor ADRB3:c.190C allele was 2.9%.

The distributions of genotypes or alleles between the two groups were not statistically significantly different, and the ADRB3:rs4994 genotype distribution in the whole studied group (iOAB + nOAB) was not consistent with the Hardy–Weinberg equilibrium (*p* < 0.001).

No statistically significant differences in age, body mass, body height, BMI, number of pregnancies, number of deliveries, or number of cesarean sections were found between women with idiopathic OAB and women with neurogenic OAB (Table S1).

There were no statistically significant differences between the iOAB and nOAB subgroups for urinary frequency, nocturia, urgency, or urgency incontinence, or for the scores of ICIQ-OAB part A, ICIQ-OAB part B, ICIQ-LUTS-QoL part A, or ICIQ-LUTS-QoL part B, before or 3 months after the injection of botulinum toxin-A, as well as for absolute or relative reductions in all symptoms or scores (Table S2 or Table S3, respectively).

There were no statistically significant differences in the frequency distribution of the score reduction greater than 50% between nOAB (*n* = 80) and iOAB (*n* = 35) women for ICIQ-OAB part A (60.0% vs. 62.5%; *p* = 0.800), ICIQ-OAB part B (71.4% vs. 77.5%; *p* = 0.485), ICIQ-LUTS-QoL part A (31.4% vs. 47.5%; *p* = 0.109), or for ICIQ-LUTS-QoL part B (68.6% vs. 80.0%; *p* = 0.184). A reduction greater than 50% in all four questionnaire parts was found in 8 (22.9%) nOAB patients and in 31 (38.7%) iOAB patients (*p* = 0.098). No score reduction was noted in 3 of 115 women for both ICIQ-OAB parts A and B, in 5 of 115 women for ICIQ-LUTS-QoL part A, and in 6 of 115 females for ICIQ-LUTS-QoL part B (Table S4).

Due to the lack of statistically significant differences between iOAB and nOAB women, we combined both subgroups and variables in a newly created group (iOAB+nOAB), which was analyzed with regard to the ADRB3 polymorphism. Because of the small number of CC homozygotes, the three ADRB3:rs4994 CC homozygous women and the seven TC heterozygotes (Table S4) were pooled for further, i.e., dominant, analyses.

The homozygous TT women and women carrying at least one C allele (TC heterozygotes or CC homozygotes) did not statistically differ significantly with regard to age, body mass, body height, BMI, number of pregnancies, number of deliveries, or number of cesarean sections (Table 1).

There were no statistically significant differences between TT homozygotes and TC + CC subjects for urinary frequency, nocturia, urgency, or urgency incontinence, or for the scores of ICIQ-OAB parts A or B or ICIQ-LUTS-QoL parts A or B, completed before and three months after the injection of botulinum toxin-A, as well as no statistically significant differences for absolute or relative reductions in all symptoms or scores (Table 2 or Table 3, respectively).

There were no statistically significant differences in the frequency distribution of score reduction greater than 50% between OAB homozygous TT (*n* = 105) patients and those carrying at least one C allele (*n* = 10) for ICIQ-OAB part A (62.9% vs. 50.0%; *p* = 0.424), ICIQ-OAB part B (75.2% vs. 80.0%; *p* = 0.737), ICIQ-LUTS-QoL part A (43.8% vs. 30.0%; *p* = 0.399), or for ICIQ-LUTS-QoL part B (74.3% vs. 100.0%; *p* = 0.114) (Table S1).

## 4. Discussion

The activity of botulinum toxin mainly affects a decrease in the pre-synaptic release of acetylcholine and temporary muscle paralysis or the inhibition of glandular secretion [21]. The specific binding of a botulinum toxin protein to a pre-synaptic membrane of a peripheral cholinergic neuron is mediated via a double interaction with a polysialoganglioside and a protein receptor [22]. Loop 4 of the luminal glycosylated domain of a synaptic vesicle protein (SV2) has been identified as the protein receptor interface for botulinum toxin-A. The light chain of botulinum toxin-A is a zinc metalloprotease that enters the nerve terminal cytosol and cleaves SNAP-25 (synaptosomal-associated 25 kDa protein) at its carboxyl terminal peptide bond Gln197-Arg198, thus blocking neurotransmitter release [23]. The findings of Carle et al. [21] and Pirazzini et al. [23] excluded the possibility that primary resistance to botulinum toxin-A is due to either mutations at the toxin-binding sites of the SV2 protein or due to mutations at the SNAP-25 cleavage site. However, Moreno-Mayordomo et al. revealed that the response to therapy with botulinum toxin-A in patients with chronic migraine was associated with single nucleotide polymorphisms located in *CALCA* or *TRPV1* genes related to migraine pathophysiology [24]. Previously, the *ADRB3*:rs4994 polymorphism has been associated either with a predisposition to OAB in non-European women [9,10] or with the efficacy of a cholinergic muscarinic receptor antagonist in OAB treatment in Turkish children [25].

The response to treatment with botulinum toxin-A in our study was measured using validated scoring instruments (ICIQ-OAB and ICIQ-LUTS-QoL). As a single administration of botulinum toxin-A leads to the temporary paralysis of human skeletal muscles lasting three to four months [23], our female patients were asked to complete the questionnaires at baseline (prior to injection) and three months after the administration of botulinum toxin.

The results of our study clearly confirmed the previous observations that the intra-detrusor injection of botulinum toxin-A in females with overactive bladder is an effective and safe treatment, capable of improving quality of life [26,27,28,29,30].

Goldman et al. indicated that, in patients with overactive bladder symptoms, the definition of response to treatment could use a threshold of 50–100% improvement in general or specific symptoms [31] and that this usage is quite common. The median improvement in ICIQ-OAB part A for our patients was higher than 60%, and there were no statistically significant differences in relative reductions (∆_%_) for part A between iOAB and nOAB women. In addition, the improvement threshold higher than 50% was exceeded in over 60% patients. The lack of statistically significant differences between iOAB and nOAB women in the frequency distributions of *ADRB3*:rs4994 variants and in the efficacy of treatment encouraged us to analyze the association in the combined group consisting of 105 women. As before, in this group, there were no statistically significant differences in the efficacy of botulinum toxin-A when women with TT homozygotes were compared with women carrying at least one C allele. In three of four questionnaire parts, the improvement was slightly (≤3.7%) higher for TC + CC women as compared to those with TT homozygotes, and for only ICIQ-OAB part A, the median of improvement (higher than 50%) for TC + CC women was approximately 10% lower than for those with TT homozygotes. In addition, only half of patients with a C allele had a score improvement for this questionnaire exceeding 50%, as compared to almost 63% of the females with two T alleles. This observation seems to be quite relevant, because previously, Gurocak et al. revealed that in Turkish children with overactive bladder, the inhibition of cholinergic activity with oxybutynin, a cholinergic muscarinic receptor antagonist, was only highly efficient in *ADRB3*:rs4994 TT homozygotes [25].

We are fully aware that the major limitation of our study that does not allow us to draw reliable conclusions is its relatively low statistical power. This statistical under-powering results mainly from a relatively small sample size and the low prevalence of the *ADRB3*:rs4994 polymorphism in our female OAB patients. By using the Open Epi (www.openepi.com), free and open-source software for epidemiologic statistics, we computed the minimum sample size for 80% statistical power and a 5% type I error rate (α), assuming a ratio of women with TT homozygotes to carriers of at least one C allele equal to 10.5 (105/10) and a frequency of score improvement (higher than 50%) for ICIQ-OAB part A equal to 62.9% in women with TT homozygotes, or 50% in women carrying at least one C allele. Under the above assumptions, the confidence interval of the minimum estimated sample size needed ranged from 1406 to 1524. We also want to emphasize that the prevalence of the *ADRB3*:c.190C allele in our cohort (5.6%) was smaller than those reported from studies carried out either on Polish women without OAB (from 8.6% to 10.3%) [32,33,34,35] or on other OAB patients of European descent (8.1%, 9.0%, and 10.8%) [13,14,36]. It is also worth noting that, to date, the association of *ADRB3*:rs4994 with a predisposition to OAB has been reported only by Ferreira et al. for 49 Brazilian females (17 white women and 32 non-white women; the frequency of *ADRB3*:c.190C allele: 25.5%) [9] and by Honda et al. for 100 Japanese females (the frequency of ADRB3:c.190C allele: 26.0%) [10]. A lack of confirmation of these results in European patients might be related to a much lower frequency of the *ADRB3*:rs4994 polymorphism in subjects of European descent than in Latin America and Japan (Additionally, for this reason, OAB risk from *ADRB3* polymorphism was not calculated in our study).

## 5. Conclusions

Our results do not support the hypothesis that the *ADRB3*:rs4994 polymorphism is associated with the response to the intra-detrusor injection of botulinum toxin-A in female patients with overactive bladder. Further studies with a larger sample size are required to solve this issue.

## Figures and Tables

**Table 1:** Basic characteristics and pre-procedure urodynamic parameters of women with overactive bladder in regard to *ADRB3*:rs4994 genotype.

Variable	*ADRB3*:rs4994 Genotype		*p*
	TT (*n* = 105)	TC + CC (*n* = 7 + 3)	TT vs. TC + CC
Age [years]	62 (22:86)	50 (30:69)	0.051
Body height [m]	164 (150:175)	163 (158:175)	0.827
Body mass [kg]	73 (47:116)	73.5 (61:85)	0.882
BMI [kg/m^2^]	27 (19:44)	28 (23:31)	0.968
Pregnancies, n	2 (0:7)	2 (1:6)	0.785
Deliveries, n	2 (0:7)	2 (1:4)	0.602
Cesarean section, n	2 (0:7)	0 (0:4)	0.457

Quantitative data are presented as median (minimum:maximum); continuous data to 2 significant figures; integer data (including body height and mass) to the nearest multiple of 0.5; and *p* values to 3 decimal places. TT = [T];[T], TC = [T];[C], CC = [C];[C].

**Table 2:** OAB symptoms in women with overactive bladder, before and three months after intra-detrusor injection of botulinum toxin-A, in regard to *ADRB3*:rs4994 genotype.

Symptoms	Time Code	*ADRB3*:rs4994 Genotype		*p*
		TT (*n* = 105)	TC + CC (*n* = 7 + 3)	TT vs. TC + CC
Frequency	0	3 (0:4)	2 (1:4)	0.575
	3	1 (0:3)	1 (0:1)	0.459
	∆_0–3_	2 (−1:4)	2 (0:4)	0.984
	∆_%_	75 (−100:100)	88 (0:100)	0.704
Nocturia	0	3 (1:4)	3 (1:4)	0.682
	3	1 (0:4)	1 (0:2)	0/849
	∆_0–3_	1 (−1:4)	1 (0:4)	0.912
	∆_%_	50 (−50:100)	42 (0:100)	0.569
Urgency	0	4 (0:4)	4 (2:4)	0.682
	3	1 (0:4)	2 (0:3)	0.849
	∆_0–3_	2 (−1:4)	3 (0:4)	0.741
	∆_%_	67 (−100:100)	63 (0:100)	0.952
Urgency incontinence	0	3 (0:4)	3 (2:4)	0.390
	3	1 (0:4)	1 (0:3)	0.548
	∆_0–3_	2 (−1:4)	2 (0:4)	0.704
	∆_%_	67 (−100:100)	58 (0:100)	0.873

Quantitative data are presented as median (minimum: maximum); continuous data to 2 significant figures; integer data to nearest multiple of 0.5; and *p* values to 3 decimal places. Time codes: 0 = before injection, and 3 = 3 months after injection; ∆_0–3_ = absolute reduction in score, and ∆_%_ = relative (percentage) reduction in score, between time codes 0 and 3. TT = [T];[T], TC = [T];[C], CC = [C];[C].

**Table 3:** ICIQ-OAB and ICIQ-LUTSqol questionnaire scores from women with overactive bladder, before and three months after intra-detrusor injection of botulinum toxin-A, in regard to *ADRB3*:rs4994 genotype.

Variable	Time Code	*ADRB3*:rs4994 Genotype		*p*
		TT (*n* = 105)	TC + CC (*n* = 7 + 3)	TT vs. TC + CC
ICIQ-OAB, part A	0	11 (6:16)	11.5 (7:16)	0.909
	3	4 (0:13)	4 (0:9)	0.870
	∆_0–3_	7 (−2:16)	6.5 (3:14)	0.980
	∆_%_	64 (−29:100)	55 (25:100)	1.000
ICIQ-OAB, part B	0	36 (13:40)	35 (22:40)	0.816
	3	8 (0:40)	5.5 (0:32)	0.585
	∆_0–3_	26 (−10:40)	26 (8:40)	0.812
	∆_%_	78 (−50:100)	82 (20:100)	0.585
ICIQ-LUTS-QoL, part A	0	56 (31:76)	59.5 (34:73)	0.616
	3	29 (16:68)	28 (17:47)	0.641
	∆_0–3_	23 (−12:57)	27.5 (12:48)	0.277
	∆_%_	46 (−32:75)	48 (27:74)	0.382
ICIQ-LUTS-QoL, part B	0	136 (36:190)	139.5 (82:189)	0.659
	3	19 (0:180)	16.5 (0:85)	0.641
	∆_0–3_	99 (−45:190)	102 (59:177)	0.275
	∆_%_	85 (−74:100)	88 (54:100)	0.551

Quantitative data are presented as median (minimum: maximum); precision is as in Table 1. Time codes: 0 = before injection, and 3 = 3 months after injection; ∆_0–3_ = absolute reduction in score, and ∆_%_ = relative (percentage) reduction in score, between time codes 0 and 3. TT = [T];[T], TC = [T];[C], CC = [C];[C]. ICIQ-OAB = the International Consultation on Incontinence Questionnaire for OAB, ICIQ-LUTS-QoL = the International Consultation on Incontinence Questionnaire-Lower Urinary Tract Symptoms-Quality of Life.

## Data Availability

Not applicable.

## Supplementary Materials

The following supporting information can be downloaded at: https://www.mdpi.com/article/10.3390/jcm11247491/s1, Table S1: Basic patient characteristics in regard to cause of overactive bladder (OAB); Table S2: OAB symptoms in women with overactive bladder, before and three months after intra-detrusor injection of botulinum toxin-A; Table S3: ICIQ-OAB and ICIQ-LUTSqol questionnaire scores of women with overactive bladder (OAB), before and three months after intra-detrusor injection of botulinum toxin-A; Table S4: Basic characteristics, OAB symptoms, and ICIQ-OAB or ICIQ-LUTSqol questionnaire scores from women with overactive bladder.
