# Supplementary material for: Functional Polymorphism in the ADRB3 Gene, Encoding the Beta-3 Adrenergic Receptor, and Response to Intra-Detrusor Injection of Botulinum Toxin-A in Women with Overactive Bladder
Source: J Clin Med. 2022 Dec 17;11(24):7491. doi: 10.3390/jcm11247491 (PMC9781921; doi:10.3390/jcm11247491)
Supplement: Supplementary file 1 [file jcm-11-07491-s001.zip › jcm-2073921-supplementary.pdf]

Table S1. Basic patient characteristics in regard to cause of overactive bladder (OAB).

Variable	All patients (n=115)	iOAB (n=80)	nOAB (n=35)	p iOAB vs nOAB
Age [years]	61 (22:86)	60 (30:85)	61 (22:86)	0.390
Body height [m]	164 (150:175)	163 (150:175)	165 (155:175)	0.065
Body mass [kg]	73 (47:116)	73 (47:116)	72 (49:98)	0.925
BMI [kg/m^2]	27 (19:44)	28 (20:44)	26 (19:39)	0.331
Pregnancies, n	2 (0:7)	2 (0:7)	2 (0:6)	0.087
Deliveries, n	2 (0:7)	2 (0:7)	2 (0:4)	0.200
Cesarean section, n	0 (0:2)	2 (0:7)	1 (0:40)	0.337

Quantitative data presented as median (minimum:maximum); continuous data to 2 significant figures; integer data (including body height and mass) to nearest multiple of 0.5; p values to 3 decimal places; iOAB = idiopathic OAB; nOAB = neurogenic OAB.

Table S2. OAB symptoms in women with overactive bladder, before and three months after intra-detrusor injection of botulinum toxin-A.

Symptoms	Time code	All patients (n=115)	iOAB (n=80)	nOAB (n=35)	p iOAB vs nOAB
Frequency	0	3 (0:4)	3 (0:4)	2 (1:4)	0.865
	3	1 (0:3)	1 (0:3)	1 (0:3)	0.857
	Δ_{0-3}	2 (-1:4)	2 (-1:4)	2 (0:4)	0.857
	$\Delta_{\%}$	75 (-100:100)	75 (-100:100)	75 (0:100)	0.920
Nocturia	0	3 (1:4)	3 (1:4)	3 (1:4)	0.968
	3	1 (0:4)	1 (0:4)	1 (0:4)	0.728
	Δ_{0-3}	1 (-1:4)	1 (-1:4)	1 (0:4)	0.849
	$\Delta_{\%}$	50 (-50:100)	50 (-50:100)	50 (0:100)	0.719
Urgency	0	4 (0:4)	4 (0:4)	4 (1:4)	1.000
	3	1 (0:4)	1 (0:4)	2 (0:4)	0.180
	Δ_{0-3}	2 (-1:4)	2 (0:4)	2 (-1:4)	0.352
	$\Delta_{\%}$	67 (-100:100)	67 (0:100)	50 (-100:100)	0.230
Urgency incontinence	0	3 (0:4)	3 (0:4)	2 (0:4)	0.158
	3	1 (0:4)	1 (0:3)	1 (0:4)	0.904
	Δ_{0-3}	2 (-1:4)	2 (-1:4)	2 (-1:3)	0.187
	$\Delta_{\%}$	67 (-100:100)	67 (-100:100)	50 (-33:100)	0.289

Quantitative data are presented as median (minimum : maximum); continuous data to 2 significant figures; integer data to nearest multiple of 0.5; p values to 3 decimal places Time codes: 0 = before injection; 3 = 3 months after injection; Δ_{0-3} = absolute reduction in score, and $\Delta_{\%}$ = relative (percentage) reduction in score, between time codes 0 and 3; iOAB = idiopathic OAB; nOAB = neurogenic OAB.

Table S3. ICIQ-OAB and ICIQ-LUTSqol questionnaire scores of women with overactive bladder (OAB), before and three months after intra-detrusor injection of botulinum toxin-A.

Variable	Time code	All patients (n=115)	iOAB (n=80)	nOAB (n=35)	p iOAB vs nOAB
ICIQ-OAB, part A	0	11 (6:16)	11 (6:16)	11 (7:16)	0.908
	3	4 (0:13)	4 (0:12)	4 (0:13)	0.479
	$\Delta_{0\text{-}3}$	7 (-2:16)	7 (-2:16)	6 (0:14)	0.485
	$\Delta_{\%}$	64 (-29:100)	64 (-29:100)	60 (0.0:100)	0.486
ICIQ-OAB, part B	0	36 (13:40)	35 (14:40)	38 (13:40)	0.451
	3	8 (0:40)	8 (0:40)	8 (0:35)	0.337
	$\Delta_{0:3}$	26 (-10:40)	26 (-10:40)	24 (3:40)	0.293
	$\Delta_{\%}$	78 (-50:100)	80 (-50:100)	72 (7.9:100)	0.189
ICIQ-LUTS-QoL, part A	0	57 (31:76)	57 (31:76)	55 (34:76)	0.834
	3	29 (16:68)	26 (16:68)	31 (17:68)	0.157
	$\Delta_{0\text{-}3}$	24 (-12:57)	25.5 (-12:57)	20 (-10:50)	0.176
	$\Delta_{\%}$	46 (-32:75)	47 (-32:75)	40 (-17:73)	0.132
ICIQ-LUTS-QoL, part B	0	136 (36:190)	139.5 (42:190)	127 (36:190)	0.535
	3	18 (0:180)	15 (0:156)	23 (0:180)	0.093
	$\Delta_{0\text{-}3}$	99 (-45:190)	108.5 (-45:190)	80 (-33:181)	0.063
	$\Delta_{\%}$	85 (-74:100)	89 (-74:100)	76 (-28:100)	0.058

Quantitative data are presented as median (minimum : maximum); continuous data to 2 significant figures; integer data to nearest multiple of 0.5; p values to 3 decimal places Time codes: 0 = before injection; 3 = 3 months after injection; $\Delta_{0\text{-}3}$ = absolute reduction in score, and $\Delta_{\%}$ = relative (percentage) reduction in score, between time codes 0 and 3; iOAB = idiopathic OAB; nOAB = neurogenic OAB. ICIQ-OAB = the International Consultation on Incontinence Questionnaire for OAB (ICIQ-OAB); ICIQ-LUTS-QoL = the International Consultation on Incontinence Questionnaire-Lower Urinary Tract Symptoms-Quality of Life.
